# Association between age and infection in patients with acute ST-elevation myocardial infarction

**DOI:** 10.1186/s43044-021-00137-w

**Published:** 2021-01-30

**Authors:** Magdy Algowhary

**Affiliations:** Department of Cardiovascular Medicine, Assiut University Heart Hospital, Assiut University, Asyut, 71515 Egypt

**Keywords:** STEMI, Bacteria, Young patients, Primary coronary intervention, Thrombus

## Abstract

**Background:**

ST-elevation myocardial infarction (STEMI) in young patients has a unique risk profile. We aimed to detect bacteria in aspirate of infarct artery in young versus old patients.

**Results:**

Aspirates of consecutive 140 patients who underwent a primary coronary intervention were taken for bacteriological, microscopical, and immunohistochemical (for bacterial pneumolysin) examinations. Their results were calculated in young (≤ 50 years) versus old (> 50 years) patients. Median age (interquartile range) was 45 (38–48) years in young (60 patients) and 59 (55–65) years in old (80 patients) patients, *p* < 0.0001. Both groups had similar baseline data except age, males, diabetes, hyperlipidemia, family history, lesion length, and ectatic vessel. Different bacteria were cultured in 11.3% of all patients involving 22.6% of young and 2.8% of old patients [hazard ratio 8.03 (95% CI 1.83–51.49), *p* = 0.002]. By multivariate analyses, age groups and leukocytic count were independent predictors of infection (bacteria and pneumolysin), *p* = 0.027 and *p* < 0.0001, respectively. Optimal cutoff value of leukocytic count was 12,250 cells/μl [ROC curve sensitivity 85.7%, specificity 86.4%, and AUC 0.97 (95% CI 0.95–1.0), *p* < 0.001]. Infection was an independent predictor of STEMI in young versus old patients, *p* < 0.001. Nevertheless, in-hospital events occurred insignificantly different and neither age groups nor infection was predictor of in-hospital events.

**Conclusions:**

Young patients had significantly higher percentage of bacteria in their infarcted artery than old patients. High leukocytic count in patients below 50 predicts infection that causes acute myocardial infarction. Antibacterial trials directed toward this group are required for secondary prevention.

## Background

Acute myocardial infarction (AMI) holds a substantial footprint on global health affecting more than 7 million individuals worldwide each year causing more than a third of deaths in developed countries annually [1]. The incidence of AMI in young population is universally low and varies according to location. In young Japanese patients, < 40 years, it ranges from 1.6% in AMI-Kyoto Multicenter-Risk Study [2] to less than 5% in Miyagi AMI Registry [3].The incidence is nearly 4% in Singapore [4], 5.8% in USA (between 25 and 44 years) [5], 1.3% in Poland (< 40 years) in PL-ACS Registry [6], 37.6% in England and Wales (between 18 and < 65 years) in MINAP Registry [7], and 23% in Egypt (< 55 years) [8]. The mortality rate after first AMI attack has declined in all age groups in Denmark [9] and in other developed countries due to improvement in prevention and treatment efforts. However, risk of recurrent hospitalization in young patients is still existing [10] posing a substantial burden on patients and health care resources [11]. Young patients, < 55 years, are at low risk to develop coronary artery disease though they may have traditional risk factors such as dyslipidemia, smoking, hypertension, diabetes mellitus, chronic kidney disease, male, family history, noncardiogenic cerebral infarction, and peripheral artery disease [12]. Lipid retention and modification is probably the main early trigger for inflammation; however, infection may play a crucial role in atherosclerosis and AMI affecting both old and young patients [13, 14]. This causative relation explains at least in part why infection triggers acute coronary syndromes after pneumococcal pneumonia [15], chlamydia pneumonia [16, 17], and in *Helicobacter pylori*-seropositive patients having interleukin-1 polymorphisms [18]. Samples obtained from atherosclerotic lesions by catheter-based atherectomy revealed existence of > 50 different species of bacteria [19] and 19 different fungal signatures [20]. Thrombus aspirates of patients with ST-elevation myocardial infarction (STEMI) revealed several oral species and chlamydia pneumoniae [21, 22]. Moreover, samples of epicardial adipose tissue surrounding coronary arteries obtained during bypass grafting from patients with acute coronary syndrome contained bacterial DNA [23] supporting the role of bacteria in the pathogenesis of atherosclerotic plaques and their disruption leading to acute coronary syndromes [24].

In our previous study, consecutive 370 patients with STEMI presented to emergency department between April 2016 and June 2017 who underwent primary coronary intervention were included [25]. STEMI was made when the following criteria were present: (1) typical anginal pain occurring within 24 h, (2) ST-segment elevation in 2 contiguous leads or new left bundle branch block, and (3) positive markers for elevated cardiac enzymes [26]. The use of aspiration catheter was left for the operator decision depending on thrombus burden. Bacteria existed in thrombus aspirates obtained from infarct-related arteries of STEMI patients.

The presenting study examined the percentage of bacteria according to age groups, young versus old STEMI patients, and whether or not it is a predictor of infarction especially in young patients.

## Methods

### Population and laboratory studies

A total of 140 aspirates were taken from 370 consecutive STEMI patients. Bacteriological, microscopical, and histopathological examinations were performed simultaneously depending on the size of thrombus aspirates. If the sample was enough, it was put into two sterile containers. The first container had sterile 2 ml of saline and was used for bacterial culture on different bacterial media, and the results were confirmed by VITEK 2 system (in 124 patients). The rest of the sample was embedded in formalin 10% inside the second container and was stained by hematoxylin and eosin for microscopical examination to detect inflammatory cells, polymorphonuclear leukocytes, and lymphocytes (in 79 patients). Histopathologic study was performed by withdrawing 2 ml of blood from the infarct-related artery to detect bacterial pneumolysin by using ELISA test (in 60 patients). Of note, each step was accomplished under strict sterile techniques to avoid bacterial contamination [25].

In this study, the results of infection markers—bacteria, pneumolysin, and inflammatory cellular infiltration—were classified according to patient age into 2 groups: young patients (YP) and old patients (OP). Age cutoff value of 50 years was selected based on receiver operating characteristic curve (ROC curve). Furthermore, for both age groups, in-hospital events, death, cerebrovascular stroke, reinfarction, lethal arrhythmias (ventricular tachycardia, ventricular fibrillation, asystole) and heart failure (Killip III and IV) were recorded. Death was recorded as cardiac and non-cardiac. Cardiac death was defined as death due to ventricular fibrillation, tachycardia, asystole, rupture ventricle, worsening heart failure, recurrent myocardial infarction, or cardiogenic shock. Non-cardiac death was defined as death due to cerebrovascular stroke, contrast-induced nephropathy, or other non-cardiac reasons. Reinfarction was diagnosed as rising of cardiac enzymes, CK, CK-MB, or cTnI above baseline level associated with chest pain and ST elevation/depression. Heart failure was defined as dyspnea because of worsening of left ventricular ejection fraction associated with rales at lung bases and auscultation of S3 heart sound in addition to impaired systolic function by echocardiography. All patients received optimal medical treatment for STEMI patients. The study was approved by the Ethical Committee of Faculty of Medicine in our University. All patients signed written consent for participation in the study, and the research was conducted ethically in accordance with the World Medical Association Declaration of Helsinki.

### Statistical analysis

Continuous variables were presented as mean ± SD in normal distributed variables and median (interquartile range, IQR) in non-normal distributed variables. Comparisons of means were done by using *T* test for normally distributed variables and Mann-Whitney *U* and Kruskal-Wallis tests for non-normal distributed variables. Categorical variables were presented as number (percentage). Comparisons between categorical variables were done by using chi-square with exact test. Multinomial logistic regression analysis using forward stepwise method was used to identify predictors of in-hospital clinical events and predictors of bacteria, bacterial pneumolysin, and infiltration while predictors of STEMI in YP versus OP were identified by using binary logistic regression analysis using forward stepwise method. ROC curve was used to identify optimal cutoff values for independent predictors (age and leukocytic count). All tests were performed by using SPSS package version 25 (IBM, New York, USA) except hazard ratio calculations which were calculated by using NCSS 2004/PASS 2002 (Utah, USA). Statistical tests were two-sided, and *p* < 0.05 was considered statistically significant.

## Results

### Clinical and laboratory results

STEMI patients treated with primary coronary intervention were 370 patients. Aspirates were obtained from 140 patients and were included in the study. Patients’ ages ranged from 22 to 90 years old. The majority of the patients, 81.4%, were between 41 and 70 years old. YP were 60 patients, 42.9%, while OP were 80 patients, 57.1%, Table 1. The median age of YP group was 45 (38–48) years, while the median age of OP group was 59 (55–65) years, *p* < 0.0001. Their clinical and catheterization data are summarized in Table 2. The 2 groups were comparable except for their ages, males, diabetes mellitus, dyslipidemia, positive family history, ectatic infarct artery, lesion length, intervention method (stenting), and stent length. YP group had significantly lower ages, diabetics, lesion length, stenting, and stent length and significantly higher males, dyslipidemia, positive family history, and ectatic vessels. Moreover, there was a trend for higher leukocytic count in the YP group.

**Table 1:** Age distribution in STEMI patients

**Age groups**	**Patients (*n*** **= 140)**
From 20 to 30 years (%)	5 (3.6)
From 31 to 40 years (%)	13 (9.3)
From 41 to 50 years (%)	42 (30)
From 51 to 60 years (%)	49 (35)
From 61 to 70 years (%)	23 (16.4)
From 71 to 80 years (%)	7 (5)
From 81 to 90 years (%)	1 (0.7)

Data presented as number (%)

*STEMI* ST-elevation myocardial infarction

**Table 2:** Patients’ clinical and catheterization data in each group

	**YP (*n*** **= 60)**	**OP (*n*** **= 80)**	p*
Male (%)	52 (86.7)	55 (68.8)	0.016
Age, years	45 (38–48)	59 (55–65)	< 0.0001
Diabetes mellitus (%)	12 (20)	31 (38.8)	0.026
Hypertension (%)	9 (15)	19 (23.8)	0.3
Smoking (%)	42 (70)	45 (56.3)	0.12
Hyperlipidemia (%)	17 (28.3)	2 (2.5)	< 0.001
Family history (%)	28 (46.7)	11 (13.8)	< 0.001
Chest pain duration, h	5 (3–7)	6 (3–9)	0.4
Ejection fraction (%)	50 (45.3–55.8)	50 (47.0–45.8)	0.4
CK-max, IU/L	2200 (1345–3234)	2168 (1464–3340)	0.6
CK-MB, μg/L	275 (201–393)	259 (200–379)	0.7
WBCs, cell/μL	8600 (5625–13175)	7580 (5805–10,200)	0.09
Door to balloon, min	35 (30–45)	35 (30–43)	0.6
Infarct related artery (%)			0.6
LAD	37 (61.7)	44 (55)	
LCX	6 (10)	7 (8.8)	
RCA	17 (28.3)	29 (36.3)	
Diseased vessels/patients (%)	2 (1–2)	2 (1–3)	0.28
Reference vessel, mm	3 (3–3.4)	3.25 (2.8–3.5)	0.15
Lesion length, mm	24.57 ± 7.91	27.56 ± 6.93	0.02
Ectatic vessel (%)	9 (15)	3 (3.8)	0.03
Thrombus grading (%)			0.28
5	35 (58.3)	52 (65)	
4	21 (35)	20 (25)	
< 4	4 (6.7)	8 (10)	
Preintervention TIMI flow (%)			0.9
≤ 1	55 (91.7)	74 (92.5)	
> 1	5 (8.3)	6 (7.5)	
Intervention method (%)			0.02
Aspiration	3 (5)	2 (2.5)	
POBA	7 (11.7)	1 (1.3)	
Stenting	50 (83.3)	77 (96.3)	
Stent type (%)			0.8
DES	13 (26)	18 (23.4)	
BMS	37 (74)	59 (76.6)	
Stent length, mm	25 (20–33)	30 (24–38)	0.04
Stent deployment pressure, atm	14 (12–16)	14 (12–16)	0.6
Postintervention TIMI flow (%)			0.5
3	57 (95)	75 (93.8)	
< 3	3 (5)	5 (6.2)	
GPIIb/IIIa inhibitors (%)	28 (46.7)	36 (45)	0.9
ST-segment resolution (%)	45 (75)	59 (73.8)	0.9

Data presented as number (%), median (IQR), or mean ± SD

*BMS* bare metal stent, *DES* drug-eluting stent, *GP IIb/IIIa* glycoprotein IIb/IIIa, *LAD* left anterior descending branch, *LCX* left circumflex branch, *OP* old patients group, *POBA* plain old balloon angioplasty, *RCA* right coronary artery, *TIMI* thrombolysis in myocardial infarction, *YP* young patients group

*YP versus OP

The results of bacterial culture in both groups are shown in Table 3. Five types of bacteria were present in the aspirates of all examined patients, and most of them were *Klebsiella pneumoniae*. Positive samples for bacteria were present in 14 patients (11.3%). Twelve of them, 85.7%, were present in YP group, 22.6%, while 2 patients were present in OP group, 2.8%, *p* = 0.001. Detailed results of bacterial culture, microscopical analysis (heavy inflammatory cell infiltration), and bacterial penumolysin are shown in Table 4. Of note, inflammatory cell infiltration was present in all studied aspirates. Mild inflammatory cell infiltration was dominant in 73.4% of all patients while heavy infiltration was present in 26.6% of all patients. Also, only 2 aspirates, 3.33%, from both groups had detectable bacterial pneumolysin. Bacteria and its product, pneumolysin, were present more significantly in YP group than in OP group (21.7% vs. 3.8%, respectively), *p* = 0.002. Hazard ratios of bacteria and bacteria or pneumolysin in YP group were significantly high [8.03 (95% CI 1.83–51.49), *p* = 0.002, and 5.78 (95% CI 1.64–25.11), *p* = 0.002, respectively].

**Table 3:** Types of bacteria in each group

	**YP (*n*** **= 53/60)**	**OP (*n*** **= 71/80)**	**All patients (*n*** **= 124/140)**
*Klebsiella pneumoniae (%)*	5 (9.4)	1 (1.4)	6 (4.8)
*Staphylococcus hominis* (%)	3 (5.7)	0 (0)	3 (2.4)
*Staphylococcus pseudintermedius* (%)	2 (3.8)	1 (1.4)	3 (2.4)
*Enterococcus bacterium* (%)	1 (1.9)	0 (0)	1 (0.8)
*Pseudomonas stutzeri* (%)	1 (1.9)	0 (0)	1 (0.8)
Total (%)	12 (22.6)	2 (2.8)	14 (11.3)

Data presented as number (%)

*OP* old patients group, *YP* young patients group

**Table 4:** Results of bacteria, pneumolysin, infiltration, and hazard ratio of young versus old patients

	**YP (*n*** **= 60)**	**OP (*n*** **= 80)**	***p***	**HR**	***p*** *
Bacteria (%)	12 (22.6)	2 (2.8)	0.001	8.03 (95% CI, 1.83–51.49)	0.002
Bacteria or pneumolysin (%)	13 (21.7)	3 (3.8)	0.002	5.78 (95% CI 1.64–25.11)	0.002
Heavy inflammatory cell infiltration (%)	8 (23.5)	13 (28.9)	0.6	0.81 (95% CI 0.34–1.86)	0.78
Bacterial pneumolysin (%)	1 (3.8)	1 (2.9)	1	1.31 (95% CI 0.04–47.56)	0.59
Bacteria or heavy infiltration (%)	18 (34)	15 (21.1)	0.12	1.61 (95% CI 0.85–3.06)	0.16
Bacteria, heavy infiltration, or pneumolysin (%)	19 (31.7)	16 (20)	0.2	1.58 (95% CI 0.85–2.98)	0.16

Data presented as number (%)

*CI* confidence interval, *HR* hazard ratio, *OP* old patients group, *YP* young patients group

*Significance of HR

### In-hospital clinical events

In-hospital clinical events were not significantly different between both groups, 7 patients, 11.7%, in YP versus 7 patients, 8.8%, in OP, *p* = 0.8. Contrast-induced nephropathy was encountered in YP group only, 2 (3.3%), while cerebrovascular stroke and heart failure were encountered in OP group only, 1 (1.3%) and 2 (2.5%), respectively. Reinfarction was seen in 1 patient of each group, 1.7% versus 1.3%, *p* = 0.99. Cardiac death due to unsuccessful treatment of ventricular fibrillation was detected in 4 patients, 6.7%, in YP versus 2 patients, 2.5%, in OP, *p* = 0.13.

### Infection as a predictor

By multivariate analyses, independent predictors of STEMI in YP versus OP were (1) infection, bacteria, and pneumolysin [Exp B 20.72 (95% CI 3.88–110.80), *p* < 0.001]; (2) hyperlipidemia [Exp B 12.38 (95% CI 2.31–66.31), *p* = 0.003]; and (3) family history [Exp B 4.27 (95% CI 1.58-11.50), *p* = 0.004]. The model could predict 79.8% correctly, and its − 2log likelihood was 121.86 with goodness of fit Hosmer test *p* = 0.42.

Predictors of in-hospital clinical events were (1) ST-segment resolution (more than 50% reduction within 90 min after intervention), −2log likelihood ratio 151.93, *p* < 0.001; (2) use of intravenous GP IIb/IIIa inhibitors, −2log likelihood ratio 139.58, *p* = 0.015; and (3) left ventricular ejection fraction, −2log likelihood ratio 114.28, *p* = 0.043. The model could predict 83.5% correctly. Of note, age groups, bacteria, bacterial pneumolysin, and leukocytic infiltration were not predictors of in-hospital clinical events.

**Fig. 1:**
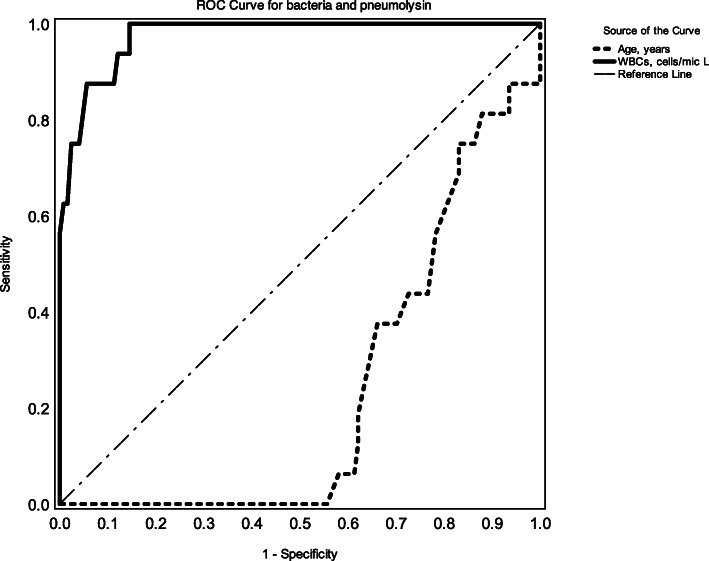
ROC curve for bacteria and pneumolysin showing age and leukocytic count predictors. The solid line represents leukocytic count. Its area under the curve is 0.97 (95% CI 0.95–1.0), *p* < 0.001. The thick dotted line represents age. Its area under the curve is 0.24 (95% CI 0.14–0.33), *p* = 0.001. The dotted line is the reference line

**Fig. 2:**
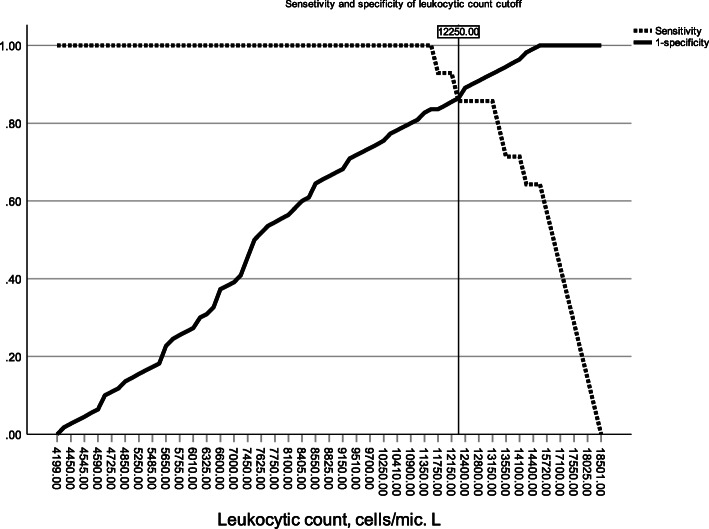
Cutoff value of leukocytic count for detection of bacteria in the aspirate of infarct related artery. It gives sensitivity of 85.7% and specificity of 86.4%

Of note, predictors of infection (bacteria, bacterial pneumolysin, and heavy infiltration) were (1) leukocytic count, −2log likelihood ratio 215.54, *p* < 0.0001; and (2) age groups, −2log likelihood ratio 86.92, *p* = 0.027. The model could predict 90.7% correctly. By using ROC curve, leukocytic count produced high area under the curve, 0.97 (95% CI 0.95–1.0), *p* < 0.001, and age groups produced a significant area under the curve, 0.24 (95% CI 0.14–0.33), *p* = 0.001. The cutoff value of 50 years gave sensitivity of 29% and specificity of 62% for detection of bacteria and penumolysin. Optimal cutoff value of leukocytic count was 12,250 cells/μl. It gave sensitivity of 85.7% and specificity of 86.4% for detection of bacteria, and sensitivity of 87.5% and specificity of 91.9% for detection of a combination of bacteria and pneumolysin (shown in Figs. 1 and 2).

## Discussion

The chief finding of this study is the presence of higher percentage of bacteria in the coronaries of young STEMI patients compared to old STEMI patients. The presence of bacteria, bacterial pneumolysin, and heavy leukocytic infiltration in coronary arteries of young STEMI patients denotes the involvement of bacteria in the process of myocardial infarction as a causative agent triggering infarction process and not responsible for in-hospital clinical events after coronary intervention. Both young-aged patients (≤ 50 years old) and a high leukocytic count (> 12250 cells/μl) are predictors of presence of bacteria in infarct-related artery.

Occurrence of myocardial infarction in very young patient, between 20 and 30 years, poses health and labor problems over a long time. Those patients are liable for heart failure, arrhythmias, recurrent angina, surgery, rehospitalization, and even recurrent infarction [11, 27]. However, revascularization techniques such as percutaneous coronary and surgical interventions has improved mortality rate in young population [28]. Of note, coronary atherosclerosis is common in elderly patients. Its incidence reported in the Framingham Heart Study above 30 years old is < 20/1000 [28, 29]. While in our study, 3.6% of AMI patients are younger, < 30 years old. This suggests different geographic distribution and more severe form in our locality. Generally, the incidence of AMI in young population, < 50 years old, is low [2–6], and it is very low in population below 40 years old, 1.6% and 1.3%, as reported in Japan and Poland, respectively [2, 6]. It may be due to different reasons and/or risk factors. Traditional risk factors such as smoking, dyslipidemia, diabetes, and obesity are not always present in every young patient with myocardial infarction. In our study, some of young STEMI patients are free from traditional risk factors; consequently, other reasons or risk factors are considered. One of the reasons widely accepted is infection theory [13, 14, 22]. This is true in our study too, where bacteria are present in 11.3% of our patients and 85.6% of them is in young group. Moreover, multivariate analysis selects bacteria as an independent predictor. The great interest of AMI in young patients tries to explain mechanisms of infarction and to discover nontraditional risk factors [30, 31]. Spontaneous coronary artery dissection, plaque erosion, coronary microvascular dysfunction, myocarditis, coronary embolism, hypercoagulability, and coronary spasm are proved to cause myocardial infarction in this group of patients. Also, drug abuse causing coronary spasm is an important mechanism. Myocardial bridge and stress-induced cardiomyopathy known as Takotsubo cardiomyopathy can explain cases with acute coronary syndrome without atherosclerotic lesions [32]. Additionally, bacterial infection can trigger coronary atherosclerosis and/or myocardial infarction which is evident in this study. YP have significantly higher males, hyperlipidemia, positive family history, and ectatic infarct-related artery while OP have higher diabetics. Similar results were reported by the AMI-Kyoto Multi-Center Risk Study Group in Japan except vessel ectasia [2] and more recently by Batra et al. [33].

In our study, 5 different types of bacteria are involved in the pathogenesis of AMI in 11.3% of patients. Higher incidence is reported by polymerase chain reaction for bacterial DNA [21, 22] supporting the role of infection in triggering acute coronary syndrome [13, 17]. Also, more diverse bacteria species are detected in atherosclerotic plaques [19, 24]. Their sources are usually different. Oral [34], periodontal [21], respiratory [15, 35–38], and gut microbiota [39–41] are involved in the pathogenesis of atherosclerosis and/or infarction. Although sieving theory, i.e., bacteremia followed by localization of bacteria at coronary artery, may theoretically explain presence of bacteria at coronary artery, absent [19] or lower concentrations [22] of bacteria in the systemic circulation exclude that theory. Bacteria and bacteria products as pneumolysin can cause direct cytotoxic effect, cellular pore-forming, activate cytokines, interleukin 6, tumor necrosis factor a, and activate neutrophil extracellular trap (NETosis) formation causing cellular damage [36]. The resultant degradation of plaque cap will eventually lead to plaque disruption and coronary thrombosis.

The in-hospital clinical outcomes in YP and OP are comparable. Neither age groups nor bacteria are predictors of short-term outcomes suggesting the role of bacteria as a triggering mechanism in the pathogenesis of myocardial infarction and no effect after primary coronary intervention. Of note, knowing the group with high percentage of bacteria, i.e., YP with elevated leukocytic count, does not only suggest nontraditional risk factors but also may guide preventive trials to select ischemic patients who may benefit from antimicrobial treatment which usually fails to give satisfactory secondary prevention results [42, 43].

## Study limitations

The study has limitations. Use of thrombus aspiration catheters was operator-dependent decision so that not all incoming patients, 370 patients, were included in the study; however, calculating the power of our sample size, 60 and 80, with the 2 proportions, 22.6% and 2.8%, gives 91% which is good. The size of aspirate from the infarct-related artery was variable so laboratory tests could not be performed for all the patients equally. Detection of bacteria by polymerization chain reaction could not be performed because of logistic obstacles. Searching for the source of bacteria was not our scope. Detection of fungal and viral microbiome could not be performed. The study is a single center study though it is a tertiary center.

## Conclusions

The aspirates of infarct-related artery contain bacteria which is present in higher concentration in young patients than old patients. Nontraditional risk factor for coronary artery disease is infection which can cause acute myocardial infarction especially in young patients and can be predicted by high leukocytic count in young patients. Further antimicrobial trials are needed focusing on this group of patient for secondary prophylaxis.

## Data Availability

The manuscript data is available on request to the corresponding author.

## Abbreviations

AMI: Acute myocardial infarction
CK: Creatine kinase
CK-MB: Creatine kinase myocardial band
DNA: Deoxyribonucleic acid
GP IIb/IIIa inhibitors: Glycoprotein IIb/IIIa inhibitors
IQR: Interquartile range
OP: Old patients (> 50 years old) with STEMI
STEMI: ST-elevation myocardial infarction
cTnI: Cardiac troponin I
YP: Young patients (≤ 50 years old) with STEMI
